# Therapeutic Effectiveness of a Novel Cranial Remolding Helmet (baby band2) for Positional Plagiocephaly: A Multicenter Clinical Observational Study

**DOI:** 10.3390/jcm13195952

**Published:** 2024-10-07

**Authors:** Nobuhiko Nagano, Risa Kato, Takanori Noto, Midori Hijikata, Aya Okahashi, Aya Nakanomori, Hiroshi Miyabayashi, Kayo Yoshikawa, Nobutaka Ichiwata, Hiroshi Saito, Mari Sasano, Koichiro Sumi, Ichiro Morioka

**Affiliations:** 1 Department of Pediatrics and Child Health, Nihon University School of Medicine, Itabashi 173-8610, Japan; nagano.nobuhiko@nihon-u.ac.jp (N.N.); kato.risa@nihon-u.ac.jp (R.K.); noto.takanori@nihon-u.ac.jp (T.N.); hijikata.midori@nihon-u.ac.jp (M.H.); okahashi.aya@nihon-u.ac.jp (A.O.); 2 Department of Pediatrics, Kasukabe Medical Center, Kasukabe 344-8588, Japan; nakanomori.aya@nihon-u.ac.jp (A.N.);; 3 Department of Pediatrics, Iino Hospital, Chofu 182-0024, Japan; kay-yoshikawa@iino-hospital.or.jp; 4 Department of Pediatrics, Kobari General Hospital, Noda 278-8501, Japan; ichiwata.nobutaka@nihon-u.ac.jp; 5 Department of Pediatrics, Itabashi Chuo Medical Center, Itabashi 174-0051, Japan; saito.hiroshi0130@gmail.com; 6 Department of Neurological Surgery, Kanagawa Children’s Medical Center, Yokohama 232-8555, Japan; sasano.mari@nihon-u.ac.jp; 7 Department of Neurological Surgery, Nihon University School of Medicine, Itabashi 173-8610, Japan; sumi.koichiro@nihon-u.ac.jp; 8 Tsuchiya Children’s Hospital, Kuki 346-0003, Japan

**Keywords:** cranial vault asymmetry index, helmet therapy, positional plagiocephaly, three dimensional data

## Abstract

This multicenter study evaluated the efficacy and safety of a novel cranial remolding helmet (baby band2), which is completely custom-made based on the shape of an infant’s cranium. The study included 224 full-term infants from moderate to very severe positional plagiocephaly in Japan. Cranial geometry was assessed before and after helmet therapy using a three-dimensional scanner, and changes in the cranial vault asymmetry index (CVAI) were analyzed. The CVAI improved significantly in all patients, with the most significant improvement observed in the most severely affected group [very severe group: −9.1, severe group: −6.6, moderate group: −4.4 (mean values), *p* < 0.001]. The group that started therapy before the age of 7 months showed greater improvement compared to those who started therapy at the age of 7 months or older; however, both groups demonstrated significant improvement (<7 months group: −6.6, ≥7 months group: −4.4 (mean values), *p* < 0.001). No significant differences were observed in therapy efficacy between the centers (*p* = 0.402) and sex (*p* = 0.131). During the study period, helmet therapy did not lead to head circumference stunting, and the incidence of redness with baby band2 was five patients (2.2%). This study demonstrated that baby band2 is effective and safe for the therapy of positional plagiocephaly.

## 1. Introduction

To reduce the incidence of sudden infant death syndrome, the American Academy of Pediatrics recommends that infants not be placed in a prone position during sleep. After the recommendation, the incidence of positional plagiocephaly increased from 0.3% to 48% [1,2,3]. Recent studies showed that severe cases of positional plagiocephaly do not resolve spontaneously [4] and can lead to complications such as strabismus [5], otitis media [6,7], impacts on psychomotor development [8,9,10,11,12,13], and misalignment of teeth during chewing [14,15]. Thus, cranial remolding helmet therapy for infants with positional plagiocephaly is becoming increasingly popular in Japan. Several cranial helmets have been approved as medical devices worldwide, and their therapeutic efficacy and safety for positional plagiocephaly have been demonstrated in Japanese studies [16,17,18].

**Figure 1:**
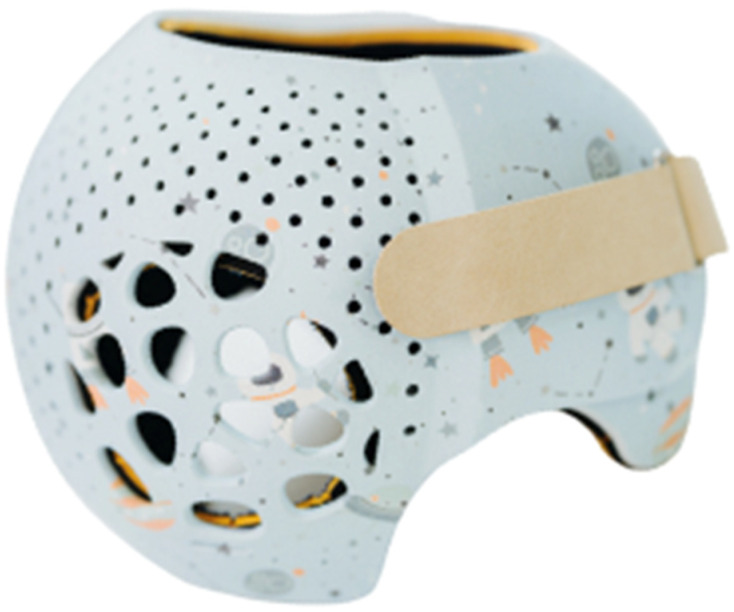
Appearance of the baby band2.

1. Baby band2 (Medical Device Approval No.: 30400BZX00252000, Figure 1) is a cranial remolding helmet developed by Berry Inc. and introduced in November 2022. This device is designed to optimize therapeutic efficacy, patient comfort, and clinical practicality simultaneously. The key features of baby band2 are as follows.
2. Customized Manufacturing: The helmet utilizes 3D printing technology for the production of individually tailored helmets optimized for each patient’s specific cranial shapes, ensuring precise treatment for various degrees of positional plagiocephaly.
3. A Single-Device Treatment: The helmet’s external structure is pre-formed to accommodate the anticipated final corrected cranial shape, enabling the completion of treatment with a single device potentially reducing overall treatment duration and cost.
4. Efficient Growth Promotion Mechanism: A proprietary internal cushioning system temporarily inhibits growth along the longitudinal axis, thereby promoting more effective growth in the flattened area.
5. Adjustment System Without Specialized Technicians: The helmet incorporates a system for adjusting the flattened area with supplementary cushioning to accommodate cranial growth, thus minimizing helmet displacement. The automated placement of additional cushioning, guided by 3D data analysis, eliminates the requirement for specialized cranial orthotic specialists.
6. Cloud-Based Data Management System: Three-dimensional cranial data are stored and managed in a cloud-based system, accessible to both clinicians and patients. This data visualization capability facilitates multi-institutional clinical management and enhances the safety and reliability of patient care.
7. Customizable Aesthetics: The helmet offers various color and pattern options for its external shell, facilitating personalization. This feature is designed to enhance patient engagement and potentially improve treatment adherence through increased personal connection to the device.

This study aimed to evaluate the efficacy and safety of a novel cranial remolding helmet (baby band2) for positional plagiocephaly using 3D data before and after therapy. This detailed evaluation of the efficacy and safety of the baby band2 therapy will enable the communication of appropriate information to patients and contribute to the standardization of this therapeutic technique.

## 2. Subjects and Methods

From 15 July 2023 to 31 May 2024, therapy with a novel cranial remolding helmet (baby band2) was started and completed for cases of moderate to severe positional plagiocephaly at the Nihon University Itabashi Hospital, Itabashi Chuo Medical Center, Tsuchiya Children’s Hospital, Kobari General Hospital, and Iino Hospital, and full-term infants who completed therapy were taken as participants. All participants underwent plain head X-rays or computed tomography before the start of helmet therapy to evaluate for craniosynostosis. No patients required surgical intervention by the end of helmet therapy.

### 2.1. 3D Cranial Shape Parameters

Stereophotogrammetry (VECTRA H2: Canfield Scientific, Parsippany, NJ, USA) was used to capture the cranium of each participant as a 3D data image, and three landmarks were established on the 3D image, as described in earlier studies [19,20]. The plane connecting the three points was set as the reference plane (Level 0), and the X- and Y-axis were determined using analysis software. The software constructed 10 equally spaced parallel sections from Level 0 to the calvaria. The height of each level was determined by dividing the height from the reference plane at Level 0 to the calvaria into 10 equal sections. A difference between the longer and shorter axial lengths of 30° to the left and right from the y-axis of the measurement plane was defined as cranial asymmetry (CA), and CA/shorter axial length (%) was defined as cranial vault asymmetry index (CVAI) [21,22]. The international diagnostic criterion for positional plagiocephaly is defined as CVAI > 3.5% [23,24]; however, in Japan, where the prevalence of positional plagiocephaly is higher than in other countries, the diagnostic criterion for positional plagiocephaly is set at CVAI > 5% [17]. To enable a comparison with past reports, we defined CVAI of >5% as normal, 5–6% as mild, 7–9% as moderate, 10–13% as severe, and ≥14% as very severe [25]. The helmet therapy was concluded when the CVAI reached the normal range or when the deformation was improved, and the family wished to end the therapy.

### 2.2. Investigation Items

Study 1: The effectiveness of a novel cranial remolding helmet (baby band2) was evaluated by examining changes in both CVAI and the severity of plagiocephaly before and after helmet therapy.
Study 2: CVAI improvement (ΔCVAI: CVAI before therapy − CVAI after therapy) was compared between the attending physicians.
Study 3: The effectiveness of helmet therapy was compared between the infants who started therapy at less than the age of 7 months and those who started therapy at the age of 7 months or later.
Study 4: We examined whether gender affects CVAI improvement.
Study 5: To determine whether helmet therapy affects head circumference by impairing growth, mean head circumference at the beginning and end of helmet therapy was compared with previously reported head circumference growth curves [26].
Study 6: The frequency of head dermatitis was investigated as a side effect of helmet therapy.
Study 7: The clinical factors affecting ΔCVAI were examined.

### 2.3. Statistical Analysis Methods

Results are expressed as number and mean ± standard deviation, and for comparisons, the chi-square test and the Mann–Whitney U test were used because the patient background is not normally distributed. In the analysis of clinical factors affecting ΔCVAI, we performed a multivariate logistic regression analysis using ΔCVAI as the dependent variable and sex, treatment duration, age at treatment initiation, and the treating doctor as explanatory variables. A significant difference was set at *p* < 0.05. All analyses were performed using JMP 14.0 (SAS Institute, Cary, NC, USA). This study was conducted with the approval of the Clinical Research Ethics Review Committee of Nihon University Itabashi Hospital (Approval number: RK-240514-11, Approval date: 15 May 2024).

## 3. Results

**Figure 2:**
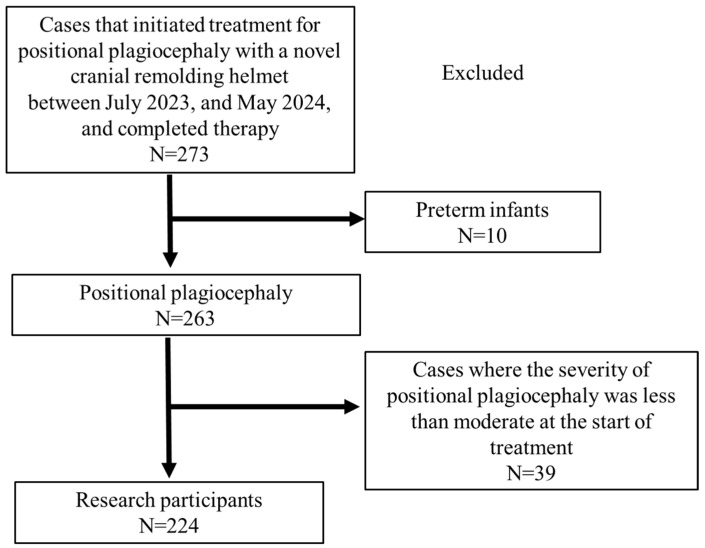
Flowchart of the enrolled infants.

Participants are shown in Figure 2. During the study period, 273 infants started and completed therapy with baby band2. Of these, 10 preterm infants with a gestational age of less than 37 weeks were excluded from the study. In addition, 39 patients with less than moderate positional plagiocephaly who underwent helmet therapy at the family’s request were excluded from the study. The total number of participants was 224, of whom 58 were treated at Nihon University Itabashi Hospital (Doctor 1), 85 at Iino Hospital (Doctor 2), and 26 at Kobari General Hospital (Doctor 3), whereas 29 at Tsuchiya Children’s Hospital and 26 at Itabashi Central General Hospital were treated by the same physician (Doctor 4). Table 1 shows the severity of patients with positional plagiocephaly treated by each doctor. There was no bias in the severity of positional plagiocephaly treated by each physician (*p* = 0.585).

### 3.1. Study 1.1: Changes in CVAI before and after Helmet Therapy

**Figure 3:**
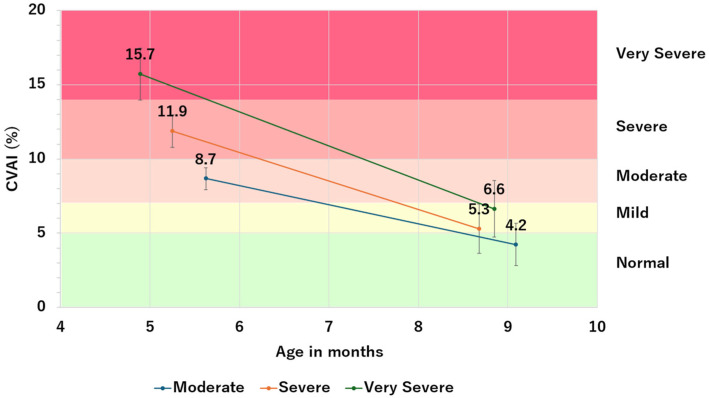
CVAI before and after helmet therapy by severity.

Figure 3 shows participants’ CVAI before and after helmet therapy, analyzed based on severity. Helmet therapy improved distortion of the head with a mean value of −9.1 ± 2.3 for the most severe group (*n* = 29), −6.6 ±1.8 for the severe group (*n* = 112), and −4.4 ± 1.4 for the moderate group (*n* = 83). Infants with higher severity had more improvement in CVAI (*p* < 0.001, Table 2). The mean duration of therapy was 4.0 months in the most severe group, 3.4 months in the severe group, and 3.5 months in the moderate group, with the duration being the longest in the most severe group (*p* = 0.028, Table 3).

#### Study 1.2: Change in Severity of Plagiocephaly before and after the Helmet Therapy

Table 4 shows the severity of plagiocephaly before and after helmet therapy. In all cases, plagiocephaly severity improved after helmet therapy (before therapy; moderate group: 83 (37%), severe group: 112 (50%), very severe group: 29 (13%), after therapy; normal group: 109 (49%), mild group: 88 (39%), moderate group: 26 (12%), severe group: 1 (0%), *p* < 0.001).

### 3.2. Study 2: Comparison of CVAI Improvement Values between Attending Physicians

**Figure 4:**
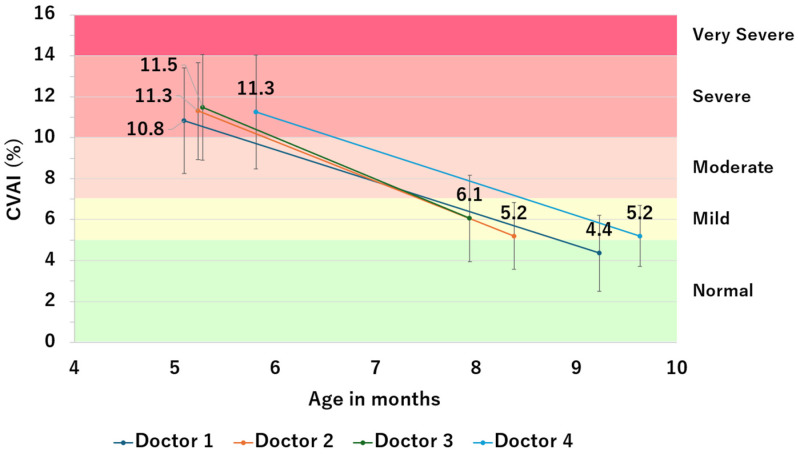
CVAI before and after helmet therapy for each physician.

Figure 4 shows CVAI before and after helmet therapy for each attending physician. CVAI improvement was noted for all physicians, although significant differences were found among the physicians (*p* < 0.001, Table 5). The duration of helmet use had a small effect on CVAI improvement; no significant difference was observed (*p* = 0.402, Table 6).

### 3.3. Study 3: Comparison of Helmet Therapy Efficacy by Age at Start of Therapy (<7 Months vs. ≥7 Months)

**Figure 5:**
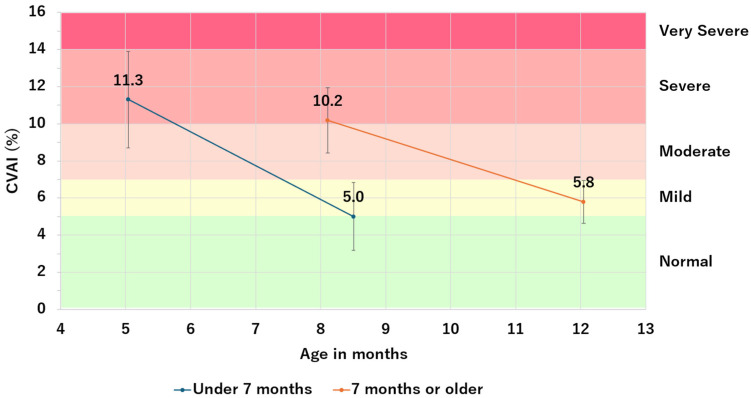
CVAI before and after helmet therapy by age in months of therapy initiation.

Figure 5 shows changes in CVAI for the group that started therapy at <7 months (*n* = 202) and the group that started at >7 months (*n* = 22). The group that started therapy at <7 months had a greater improvement in CVAI, to −6.3 ± 2.3 after therapy, while the >7 months group improved to −4.4 ± 1.6 (*p* < 0.001, Table 7). The mean duration of therapy was an average of 3.5 months for the <7 months group and 3.9 months for the >7 months group (*p* = 0.043, Table 8).

### 3.4. Study 4: Impact of Sex on CVAI Improvement

**Figure 6:**
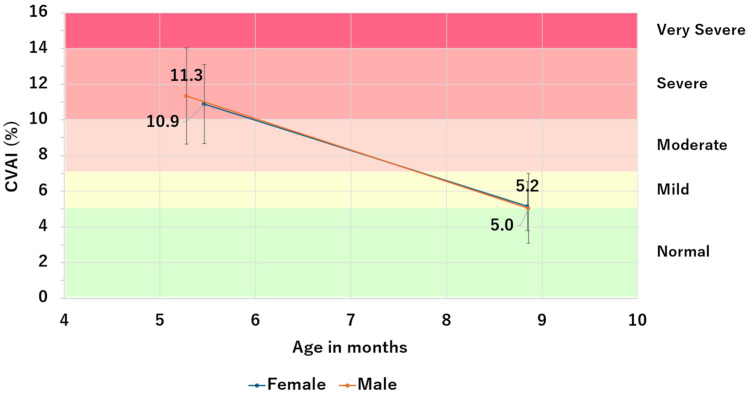
CVAI before and after helmet therapy by sex.

Figure 6 shows a comparison of changes in CVAI by sex. Regarding the effectiveness of therapy, no clear difference was observed between females (*n* = 76) and males (*n* = 148) (*p* = 0.131, Table 9). No significant difference was the therapy duration (*p* = 0.343, Table 10).

### 3.5. Study 5: Effects of Helmet Therapy on Head Circumference Stunting

**Figure 7:**
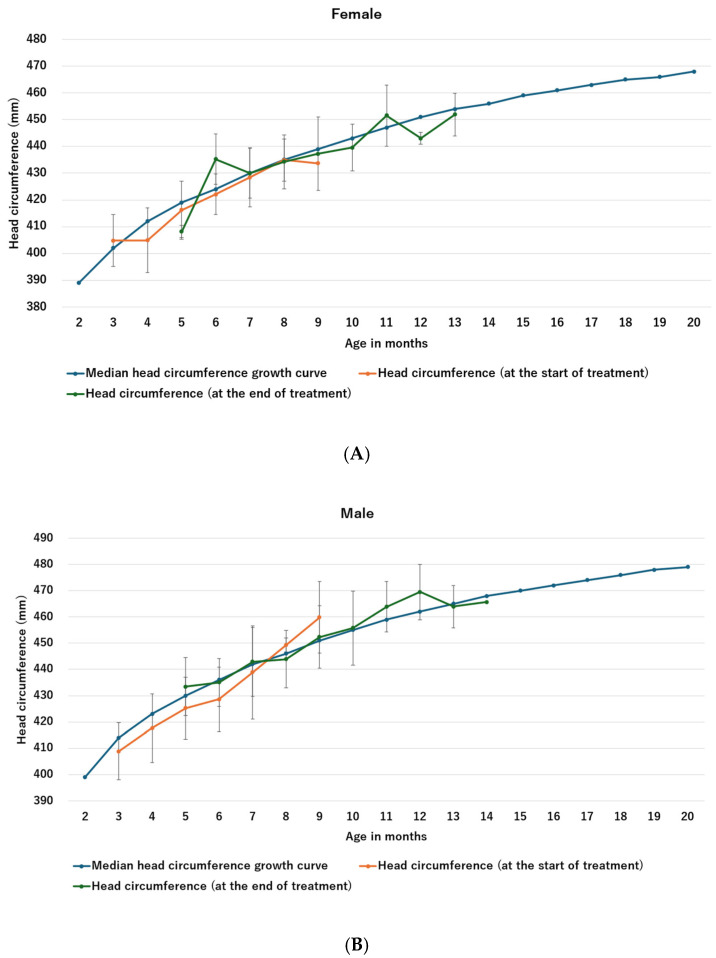
(**A**) Head circumference growth curve vs. head circumference growth curve during helmet therapy (Females). The head circumference growth curve was cited from [24]. (**B**) Head circumference growth curve vs. head circumference growth curve during helmet therapy (Males). The head circumference growth curve was cited from [24].

The mean head circumferences at the beginning and end of helmet therapy for females (*n* = 76) and males (*n* = 148) are shown in Figure 7A and Figure 7B, respectively. As compared to previously reported head circumference growth curves, no obvious head circumference stunting was observed in either group.

### 3.6. Study 6: Frequency of Dermatitis Due to Helmet Therapy

Redness of the skin on the head may occur as an adverse reaction due to chafing and pressure caused by wearing a helmet. During the study period, the incidence of redness with baby band2 was five patients (2.2%). In all cases, redness improved with temporary suspension of therapy, application of topical agents, and additional cushioning.

### 3.7. Study 7: Examination of Clinical Factors Affecting ΔCVAI

In multivariate logistic regression analysis using sex, age at treatment initiation, treatment duration, and the treating doctor, age at treatment initiation was an independent determinant factor for ΔCVAI (Table 11).

## 4. Discussion

This study found the novel cranial remolding helmet (baby band2) to be effective in improving positional plagiocephaly from moderate to higher severity. Moreover, baby band2 exhibited the following characteristics: (1) a short therapy period; (2) a certain level of efficacy even when therapy is started after 7 months of age; and (3) a little difference in efficacy among attending physicians. Conversely, although no head circumference stunting was observed, skin redness occurred in 2.2% of the patients.

### 4.1. Improvement of CVAI

Studies examining the effects of helmet therapy for positional plagiocephaly in Japan have concluded that helmet therapy is effective in improving cranial distortion [16,17,18]. Additionally, our study found no gender difference in the degree of improvement, with greater improvement in participants with more severe initial deformity and those who received therapy at a younger age. One infant’s positional plagiocephaly severity improved only from “most severe” to “severe” after helmet therapy, likely due to wearing the helmet for an average of ~7.6 h per day, significantly less than the recommended 23 h per day. For infants with a high degree of distortion, starting therapy at a younger age, after confirming they can hold their heads up, is recommended.

### 4.2. Treatment Period

Previous studies have reported varying durations of helmet therapy, ranging from an average of 6.3 months [27], approximately 3–6 months [16], an average of 21.2 weeks (standard deviation 5.3 weeks) [17], an average of 4.3 months [28], and an average of 22.4 weeks (standard deviation 6.0 weeks) [18]. Although it is difficult to make a direct comparison due to variations in treatment severity and the degree of improvement sought, the baby band2 used in this study showed a mean duration of 3.5 months (standard deviation of 1.1 months), potentially allowing for a shorter treatment period than previously reported.

### 4.3. Starting Age of Therapy

Previous reports have shown that earlier initiation of therapy, determined by age in months, results in more effective outcomes [18]. Many experts recommend starting helmet therapy at 4 to 6 months of age [29]. However, some studies suggest prioritizing repositioning and tummy time for younger infants, as these approaches can sometimes improve the condition without helmet therapy [12,30]. In addition, because physical approaches to positional plagiocephaly have also been reported to be more effective the younger the infant is [31], there are cases where helmet therapy cannot be started at 4 to 6 months of age.

The baby band2 has been shown to improve CVAI by an average of −4.4 even when therapy started after 7 months of age, indicating effectiveness in cases where the start of helmet therapy was delayed following unsuccessful repositioning and physical approaches to reduce distortion.

### 4.4. Limitations

This study has several limitations. One limitation is that the exact duration of helmet wear was not accurately recorded. Additionally, the study did not investigate the recurrence of deformation after the completion of helmet therapy. Furthermore, there have been reports indicating no significant difference in the improvement in CVAI between groups that underwent active physical therapy and those that received helmet therapy [12]. Since this study did not include a control group, further investigation through prospective studies is needed. Moreover, as the hygienic aspects of helmet therapy were not evaluated, additional assessment of the satisfaction levels of the subjects and their families is necessary.

## 5. Conclusions

Helmet therapy with baby band2 significantly improved CVAI in all cases, with the most significant improvement observed in the most severely affected group. Furthermore, a certain level of effectiveness was achieved even when therapy was started after 7 months of age, with a slightly shorter treatment duration compared to other helmet therapies. Additionally, little difference was observed in therapeutic effectiveness between medical institutions and physicians.

## Figures and Tables

**Table 1:** Distribution of severity *.

	Moderate	Severe	Very Severe	Total
Hospital 1	23	31	4	58
Hospital 2	27	46	12	85
Hospital 3	10	12	4	26
Hospital 4	23	23	9	55
Total	83	112	29	224

Data are shown as numbers. * There was no bias in the severity of positional plagiocephaly among the hospitals (*p* = 0.585).

**Table 2:** Pre- and post-therapy CVAI by severity.

Severity	Number of Patients	Pre-Therapy CVAI			Post-Therapy CVAI			ΔCVAI		
		Mean	SD	Variance	Mean	SD	Variance	Mean	SD	Variance
Moderate	83	8.7	0.7	0.5	4.2	1.4	1.9	−4.4	1.4	1.9
Severe	112	11.9	1.1	1.2	5.3	1.6	3.2	−6.6	1.8	3.2
Very Severe	29	15.7	1.8	3.1	6.6	1.9	5.4	−9.1	2.3	5.4
Total	224	11.2	2.5	6.5	5.1	1.8	5.3	−6.1	2.3	5.3

CVAI; Cranial vault asymmetry index, SD; Standard deviation.

**Table 3:** Duration of therapy by severity.

Severity	Number of Patients	Age at Therapy Initiation	Age at Therapy Completion	Therapy Duration (Months)	*p*-Value
Moderate	83	5.6 ± 1.4	9.1 ± 1.9	3.5 ± 1.3	0.028
Severe	112	5.3 ± 1.2	8.7 ± 1.8	3.4 ± 1.0	
Very Severe	29	4.9 ± 0.8	8.8 ± 1.4	4.0 ± 0.9	
Total	224	5.3 ± 1.3	8.9 ± 1.8	3.5 ± 1.1	

Data are shown as mean ± standard deviation.

**Table 4:** Change in severity before and after therapy.

		After Therapy					
		Normal	Mild	Moderate	Severe	Very Severe	Total
**Before Therapy**	Moderate	55	28	0	0	0	83
	Severe	48	49	15	0	0	112
	Very Severe	6	11	11	1	0	29
	Total	109	88	26	1	0	224

Data are shown as numbers.

**Table 5:** Differences in therapy duration by treating physician.

	Number of Patients	Age at Therapy Initiation	Age at Therapy Completion	Therapy Duration (Months)	*p*-Value
Doctor 1	58	5.1 ± 1.1	9.2 ± 1.9	4.1 ± 1.4	<0.001
Doctor 2	85	5.2 ± 1.2	8.4 ± 1.6	3.1 ± 0.8	
Doctor 3	26	5.3 ± 1.3	7.9 ± 1.6	2.7 ± 0.9	
Doctor 4	55	5.8 ± 1.3	9.6 ± 1.6	3.8 ± 0.9	
Total	224	5.3 ± 1.3	8.9 ± 1.8	3.5 ± 1.1	

Data are shown as mean ± standard deviation.

**Table 6:** Differences in therapy efficacy by treating physician.

	Number of Patients	Pre-Therapy CVAI			Post-Therapy CVAI			ΔCVAI		
		Mean	SD	Variance	Mean	SD	Variance	Mean	SD	Variance
Doctor 1	58	10.8	2.6	6.7	4.4	1.8	7.4	−6.5	2.7	7.4
Doctor 2	85	11.3	2.4	5.6	5.2	1.6	4.5	−6.1	2.1	4.5
Doctor 3	26	11.5	2.6	6.6	6.1	2.1	3.5	−5.4	1.9	3.5
Doctor 4	55	11.3	2.8	7.7	5.2	1.5	5.0	−6.1	2.2	5.0
Total	224	11.2	2.5	6.5	5.1	1.8	5.3	−6.1	2.3	5.3

CVAI; Cranial vault asymmetry index, SD; Standard deviation.

**Table 7:** Differences in therapy effects by age of therapy initiation.

Age at Therapy Initiation	Number of Patients	Pre-Therapy CVAI			Post-Therapy CVAI			ΔCVAI		
		Mean	SD	Variance	Mean	SD	Variance	Mean	SD	Variance
Under 7 months	202	11.3	2.6	6.7	5.0	1.8	5.2	−6.3	2.3	5.2
7 months or older	22	10.2	1.8	3.1	5.8	1.2	2.6	−4.4	1.6	2.6
Total	224	11.2	2.5	6.5	5.1	1.8	5.3	−6.1	2.3	5.3

CVAI; Cranial vault asymmetry index, SD; Standard deviation.

**Table 8:** Differences in therapy duration by age of therapy initiation.

Age at Therapy Initiation	Number of Patients	Age at Therapy Initiation	Age at Therapy Completion	Therapy Duration (Months)	*p*-Value
Under 7 months	202	5.0 ± 0.9	8.5 ± 1.5	3.5 ± 1.2	0.043
7 months or older	22	8.1 ± 0.9	12.0 ± 1.2	3.9 ± 0.9	
Total	224	5.3 ± 1.3	8.9 ± 1.8	3.5 ± 1.1	

Data are shown as mean ± standard deviation.

**Table 9:** Differences in therapy effectiveness by sex.

Sex	Number of patients	Pre-Therapy CVAI			Post-Therapy CVAI			ΔCVAI		
		Mean	SD	Variance	Mean	SD	Variance	Mean	SD	Variance
Female	76	10.9	2.2	4.9	5.2	1.4	4.1	−5.7	2.0	4.1
Male	148	11.3	2.7	7.3	5.0	2.0	5.8	−6.3	2.4	5.8
Total	224	11.2	2.5	6.5	5.1	1.8	5.3	−6.1	2.3	5.3

CVAI; Cranial vault asymmetry index, SD; Standard deviation.

**Table 10:** Differences in therapy duration by sex.

Sex	Number of Patients	Age at Therapy Initiation	Age at Therapy Completion	Therapy Duration (Months)	*p*-Value
Female	76	5.5 ± 1.2	8.9 ± 1.7	3.4 ± 1.1	0.343
Male	148	5.3 ± 1.3	8.9 ± 1.8	3.6 ± 1.2	
Total	224	5.3 ± 1.3	8.9 ± 1.8	3.5 ± 1.1	

Data are shown as mean ± standard deviation.

**Table 11:** Multivariate analysis.

Factor	Logarithmic Worth (95% Confidence Interval)	*p*-Value
Age at treatment initiation	8.117 (0.48–0.94)	<0.001
Sex	0.743 (−0.50–0.10)	0.181
Treatment duration	0.661 (−0.45–0.10)	0.218
Doctor	0.270 (−0.70–0.37)	0.537

## Data Availability

No new data were created or analyzed in this study. Data sharing is not applicable to this article.
